# The pathology of central nervous system inflammatory demyelinating disease accompanying myelin oligodendrocyte glycoprotein autoantibody

**DOI:** 10.1007/s00401-020-02132-y

**Published:** 2020-02-11

**Authors:** Romana Höftberger, Yong Guo, Eoin P. Flanagan, A. Sebastian Lopez-Chiriboga, Verena Endmayr, Sonja Hochmeister, Damir Joldic, Sean J. Pittock, Jan Mendelt Tillema, Mark Gorman, Hans Lassmann, Claudia F. Lucchinetti

**Affiliations:** 1 Division of Neuropathology and Neurochemistry, Department of Neurology, Medical University of Vienna, Vienna, Austria; 2 Department of Neurology, Mayo Clinic, Rochester, MN USA; 3 Department of Laboratory Medicine and Pathology, Mayo Clinic, Rochester, MN USA; 4 Department of Neurology, Medical University Graz, Graz, Austria; 5 Department of Neurology, Krankenanstalt Rudolfstiftung, Vienna, Austria; 6 Department of Neurology, Boston Children’s Hospital, Boston, MA USA; 7 Center for Brain Research, Medical University of Vienna, Vienna, Austria

**Keywords:** Demyelination, MOG, Multiple sclerosis, Biopsy, Autopsy, Acute disseminated encephalomyelitis

## Abstract

We sought to define the pathological features of myelin oligodendrocyte glycoprotein (MOG) antibody associated disorders (MOGAD) in an archival autopsy/biopsy cohort. We histopathologically analyzed 2 autopsies and 22 brain biopsies from patients with CNS inflammatory demyelinating diseases seropositive for MOG-antibody by live-cell-based-assay with full length MOG in its conformational form. MOGAD autopsies (ages 52 and 67) demonstrate the full spectrum of histopathological features observed within the 22 brain biopsies (median age, 10 years; range, 1–66; 56% female). Clinical, radiologic, and laboratory characteristics and course (78% relapsing) are consistent with MOGAD. MOGAD pathology is dominated by coexistence of both perivenous and confluent white matter demyelination, with an over-representation of intracortical demyelinated lesions compared to typical MS. Radially expanding confluent slowly expanding smoldering lesions in the white matter as seen in MS, are not present. A CD4+ T-cell dominated inflammatory reaction with granulocytic infiltration predominates. Complement deposition is present in all active white matter lesions, but a preferential loss of MOG is not observed. AQP4 is preserved, with absence of dystrophic astrocytes, and variable oligodendrocyte and axonal destruction. MOGAD is pathologically distinguished from AQP4-IgG seropositive NMOSD, but shares some overlapping features with both MS and ADEM, suggesting a transitional pathology. Complement deposition in the absence of selective MOG protein loss suggest humoral mechanisms are involved, however argue against endocytic internalization of the MOG antigen. Parallels with MOG-EAE suggest MOG may be an amplification factor that augments CNS demyelination, possibly via complement mediated destruction of myelin or ADCC phagocytosis.

## Introduction

Myelin Oligodendrocyte Glycoprotein (MOG) is located on the surface of central nervous system (CNS) myelin and oligodendrocytes and is a marker of oligodendrocyte maturation [6]. It may act as a cell adhesion molecule, a regulator of microtubule stability, and a mediator of myelin and immune interactions [32]. MOG is a part of the immunoglobulin superfamily with an extracellular domain, a transmembrane domain, a cytoplasmic loop, and a cytoplasmic tail [32, 55]. Its location on the surface of myelin led to initial speculation of its immunogenicity in multiple sclerosis (MS) but results were inconsistent, reflecting detection of non-conformational MOG epitopes and older generation assay techniques (enzyme-linked-immunosorbent-assay [ELISA]) [3]. Initial work using assays with MOG in its native conformational form gave the first clue that MOG-IgG may represent a biomarker of an inflammatory CNS demyelinating disease distinct from MS [53]. This was followed by the use of improved cell-based assay techniques using HEK 293 cells transfected with the correctly folded MOG. MOG-IgG has been shown in multiple studies from independent researchers to be a specific serum biomarker of a subset of central nervous system (CNS) inflammatory demyelinating diseases (IDD), clinically distinct from MS and aquaporin-4 (AQP4) antibody (Ab) seropositive neuromyelitis optica spectrum disorder (NMOSD) [9, 29, 34, 45, 51, 53, 62, 63]. Clinical attacks are typically more severe than in MS, with relapses often rendering a patient blind from optic neuritis (ON), wheelchair-dependent from transverse myelitis (TM) or encephalopathic from an ADEM-like presentation [7, 15, 45]. However, recovery is better than with AQP4 Ab seropositive NMOSD [15]. MOG-antibody associated disorders (MOGAD) may follow a relapsing or monophasic course. Children are predisposed and more likely to have a monophasic course [23, 29, 31, 34, 76]. Clinical diagnostic criteria have been proposed requiring one or more of ON, TM, ADEM or other brain or brainstem involvement suggestive of demyelination, MOG-IgG seropositivity and exclusion of other etiologies [45]. The pathophysiology of MOGAD has not been well elucidated. Currently, pathological studies are limited to case reports, some of which have suggested an overlap with pattern II MS pathology characterized by deposition of complement and immunoglobulin in active lesions [13, 21, 25–27, 35, 66, 67, 75]. Defining the immunopathologic features of MOGAD is necessary to improve understanding of its pathogenesis, which is a potential first step towards developing novel treatments. Herein, we describe an international collaborative biopsy and autopsy series of MOGAD.

## Materials and methods

### Patient identification and inclusion criteria

This study was approved by the Institutional Review Board of Mayo Clinic, Rochester, MN (IRB 2067-99). Patients were identified through the CNS inflammatory demyelinating disease pathologic biobanks of: the Medical University of Vienna (Austria), 6; and Mayo Clinic (Rochester, Minnesota, USA), 18. Inclusion criteria were: (1) positive MOG-IgG in serum by live cell-based assay with full length MOG in its native conformational form; (2) CNS biopsy/autopsy available; (3) sufficient archival tissue for pathological analysis [28, 45]. In total, 2 autopsies and 22 biopsies (45 blocks) met inclusion criteria. Three patients in this series were included in prior case reports [35, 39, 67].

### Neuropathological evaluation

Formalin-fixed paraffin-embedded 5 µm thick sections were stained with hematoxylin and eosin (H&E), Luxol fast blue and periodic acid Schiff (LFB/PAS), and modified Bielschowsky silver impregnation. Immunohistochemistry was performed with the avidin–biotin-complex method as previously reported, using primary antibodies against glial fibrillary acidic protein (GFAP, 1:100, DAKO, Denmark), neurofilament (1:800, steam antigen retrieval with citric acid buffer pH 6.0, DAKO, Denmark), AQP4 (1:250, Sigma-Aldrich, USA), myelin proteolipid protein (PLP, 1:500, Serotec, Oxford, UK), myelin associated protein (MAG, 1:1000, Abcam), myelin oligodendrocyte glycoprotein (MOG, 1:1000, Abcam), 2′,3′-cyclic-nucleotide 3′-phosphodiesterase (CNPase, 1:2000, Sternberger), CD3 (1:500, Leica Biosystems), CD4 (1:100, DAKO, Denmark), CD8 (1:50, DAKO, Denmark), CD68 KP1 (1:100, DAKO, Denmark), complement C9 neo-antigen (C9neo, monoclonal B7 and polyclonal, 1:200, from Professor Paul Morgan, Cardiff, UK).

The pattern of white matter demyelination was characterized as perivenous, coalescent, or confluent based on published criteria [81]. White matter lesions were staged according to demyelinating activity and immunopattern classified based on published criteria [46]. Active white matter plaque types were further defined as either chronic active plaques characterized by radially expanding lesions with a sharp myelin-laden macrophage border, or slowly expanding (smoldering lesions), with prominent microglial activation at the plaque border, and few myelin-laden macrophages. The activity stages were defined by the presence of different myelin degradation products within myeloid cells: (1) early active demyelination with myelin–laden macrophages immunoreactive for both minor (MOG, MAG) and major myelin proteins (PLP); (2) late active demyelination with macrophages immunoreactive for major myelin proteins only; (3) Inactive demyelination was absent of myelin-laden macrophages; (4) periplaque white matter (PPWM) was non-demyelinated white matter around the demyelinating plaque [5]. Cortical demyelination was classified as subpial (confluent demyelination extending from pia to the deeper cortex), intracortical (small focal perivenous demyelinating lesions within the cortex), and leukocortical (confluent demyelination involving both cortex and adjacent white matter at the cortex/white matter junctions without superficial cortex involvement). The pattern and number of cortical demyelinating plaques were determined [47], and compared with a MS pathological cohort of early MS [47].

The pattern (perivascular or parenchymal) and nature of inflammation involving the leptomeninges, cortex, deep gray matter and white matter were quantified semiquantitatively (none, mild, moderate, or marked). Perivascular lymphocytic infiltration was graded as none, mild (≤ 2 lymphocytic cuffs), moderate (3–4 cuffs), and marked (> 4 cuffs). In a subset of lesions (*n* = 7), the number of perivascular CD4 and CD8-positive lymphocytes were counted based on selecting the most inflamed vessel per tissue block and a perivascular CD4/CD8 ratio was calculated. Granulocyte perivascular and parenchymal infiltration based on total eosinophil or neutrophil number per high power microscopic field (HPF) was defined as: mild (≤ 3 cells/HPF); moderate (4–10 cells/HPF); or marked (> 10 cells/HPF). Data was reported as median and range for continuous variables and frequencies and percentages for categorical variables as appropriate.

## MOG-antibody assessment

The MOG Ab is of IgG1 isotype and positivity was confirmed and end-titers determined using serum samples analyzed with a live-cell-based fluorescence activated cell sorting (FACS) assay with full length MOG in its conformational form either at the Mayo Clinic Neuroimmunology Laboratory (Rochester, MN, USA) with a cut off for positivity of an IgG binding index of ≥ 2.5, or the Division of Neuropathology and Neurochemistry at the Medical University of Vienna (Austria) with a cut off for positivity of > 1:160 as previously described [24, 45]. Persistent seropositivity was defined as a positive MOG Ab (≥ 6 months after onset) or serial samples seropositive ≥ 6 months apart.

## Clinical data collection

Clinical details were collected by (R.H., E.P.F., A.S.L.C.) and obtained from the relevant electronic medical record by retrospective chart review at each institution along with clinical records shared by outside facilities. Detailed clinical histories are presented for both autopsy cases. For the biopsy cohort we recorded details of the demographics (age, sex), clinical phenotype of relapses including transverse myelitis [15], optic neuritis [7], ADEM-like brain presentation [45, 81], and NMOSD [79] or combinations thereof. For those with other presentations we included descriptions of their clinical manifestations. Acute and maintenance immunotherapy treatments administered were recorded. Disability based on expanded disability status scale (EDSS) score was determined at last follow-up [38]. Cerebrospinal fluid (CSF) was analyzed for white blood cell count, protein and oligoclonal bands. Retrospective review of magnetic resonance imaging of the brain was performed on 12 biopsy cases with available scans, or the written interpretation of the radiologist recorded when not available (4 biopsy cases). Lesion characteristics, location, and pattern of gadolinium enhancement were determined.

## Results

### Autopsy 1

**Fig. 1:**
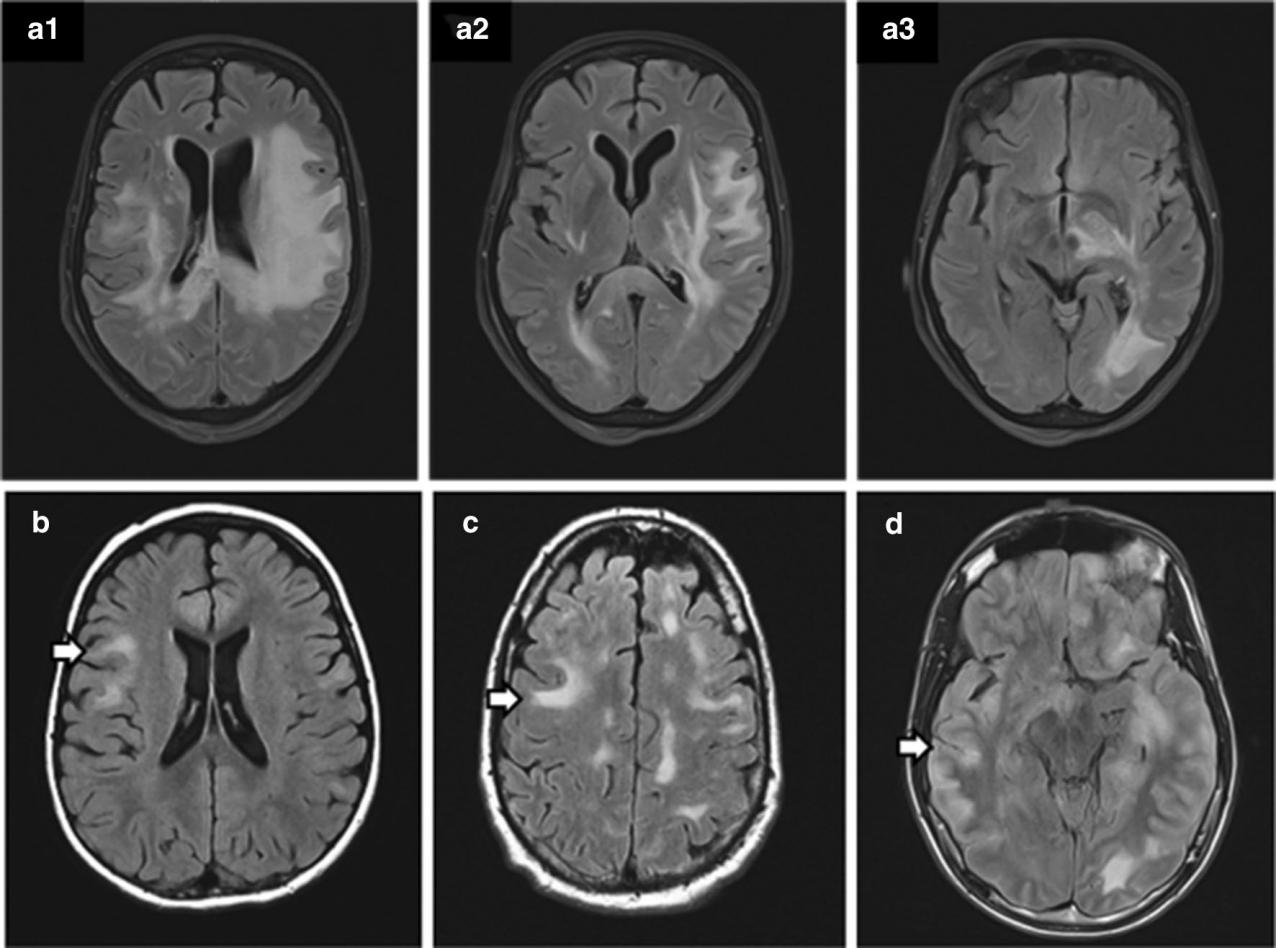
MRI findings in patients with MOG-associated encephalomyelitis. **a1–a3** Brain MRI from autopsy patient 1, obtained on day 40 after symptom onset, shows extensive, confluent T2 abnormalities involving the subcortical white matter of the frontal, temporal, and occipital lobe, bilaterally, with contrast enhancement in one fronto-parietal lesion (not shown) and extension into deep grey matter. **b–d** MRI of 3 MOGAD biopsied cases just prior to surgery with the arrow reflecting the site of biopsy

A 67-year-old woman presented with encephalopathy, dysarthria, and hemiparesis 2 weeks following a respiratory infection. The patient progressed to status epilepticus and respiratory insufficiency. Brain MRI showed extensive white matter lesions in both hemispheres, with contrast enhancement of a right fronto-parietal lesion. CSF analysis revealed 33 white blood cells (normal, 0–5) with a lymphocytic predominance and negative oligoclonal bands. After no improvement with intravenous steroids, a brain biopsy of the right frontal lesion was performed and showed multiple small fragments of CNS white matter tissue with perivenous areas of active demyelination, perivascular macrophages and mild inflammatory T cell dominated infiltrates, pathologically suggestive of ADEM (data not shown). MOG Abs were later confirmed to be positive (Titer, 1:320) and AQP4 Abs were negative. Intravenous methylprednisolone and plasma exchange were administered without clinical improvement and repeat MRI showed progression of the bi-hemispheric white matter lesions (Fig. 1a–c). Rituximab was initiated, but the patient developed sepsis and died of cardio-respiratory failure 3 weeks later, 2 months after disease onset.

**Fig. 2:**
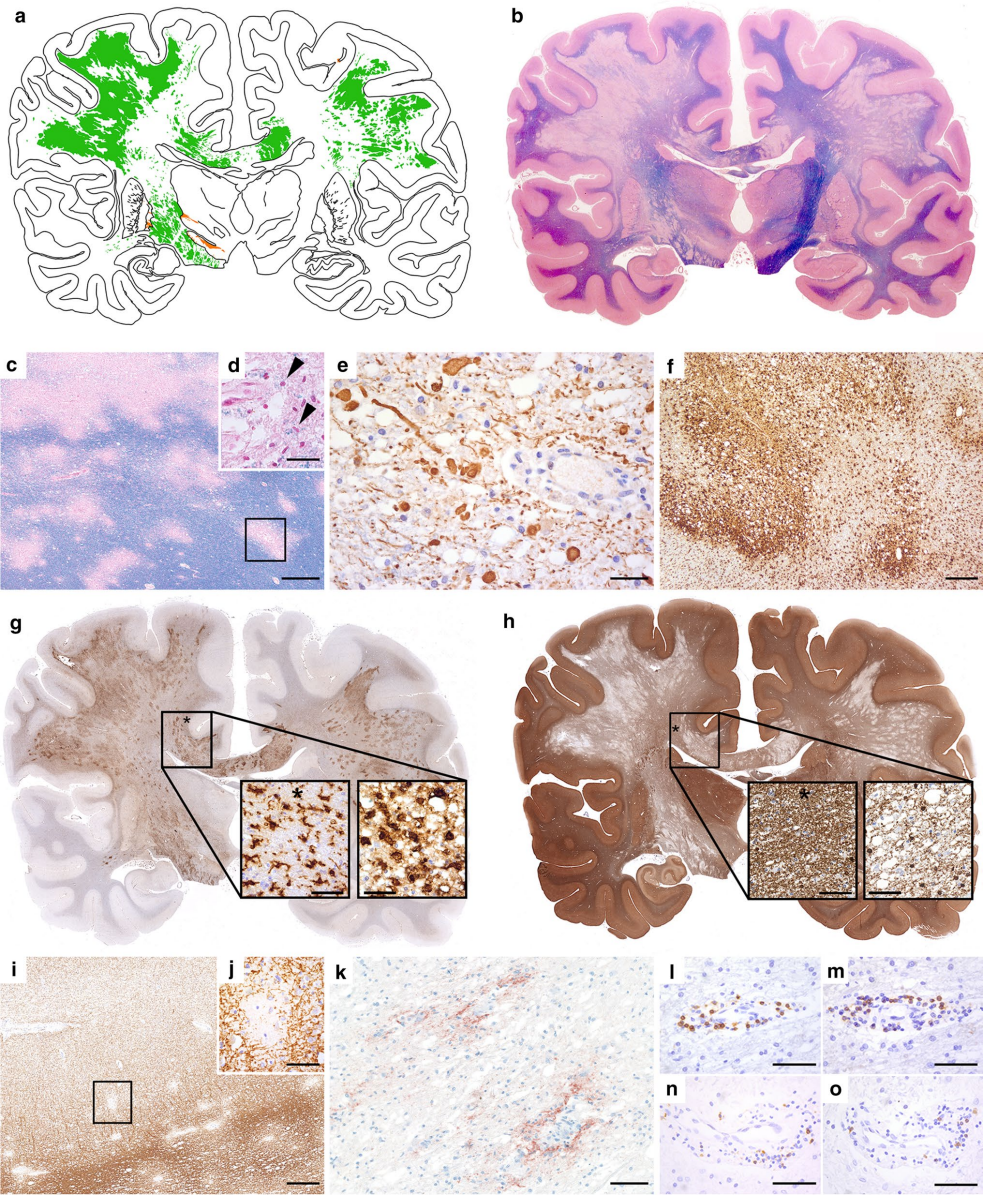
Neuropathology of autopsy case 1. Topographic evaluation shows prominent bi-hemispheric demyelination in the white matter, focally extending into the cortex and deep grey matter (**a**, green: white matter demyelination, orange: grey matter demyelination; **b**, LFB). The demyelinated lesions are partly confluent with massive perivenous accentuation (**c**, LFB) and characterized by early active demyelination with LFB-positive degradation products within the macrophage cytoplasms (**d**, arrow heads; LFB). Axons are moderately reduced and form numerous axonal spheroids (**e**, SMI31). Some of the confluent demyelinating lesions show a transition between perivascular accentuation to a rim of activated macrophages and microglia at the edge (**f**, HLADR), profound microglia activation is also seen in the periplaque white matter (**g**, asterisk in PPWM; microglia in PPWM enlarged in left image; macrophages in plaque enlarged in right image; HLADR). Within the demyelinated lesions a higher number of oligodendrocytes is visible compared to NAWM (**h**, asterisk in NAWM; oligodendrocytes in NAWM enlarged in left image, plaque enlarged in right image; TPPP/p25). Small intracortical perivenous demyelination is also present in the cortex (**i, j**; rectangle in **i** enlarged). Within the lesions profound deposition of activated complement complex is visible (**k**, C9neo antigen). The inflammatory reaction is characterized by perivascular cuffs of moderate numbers of CD3 + (**l**), CD4 + (**m**), and CD8 + T cells (**n**), and only few CD79a + B cells (**o**). Scale bars **c** 600 μm; **d, h, j** 60 μm; **i** 380 μm; **k** 100 μm; **e, g, l–o** 50 μm; **f** 300 μm

Postmortem examination of entire brain hemispheric sections showed widespread bi-hemispheric areas of demyelination in the cerebral white matter (Fig. 2a, b) characterized by multiple perivenous and coalescent lesions giving rise to confluent inflammatory demyelinating areas (Fig. 2c). All lesions showed early active demyelinating activity, characterized by LFB- (Fig. 2d) and MOG-positive myelin degradation products within macrophage cytoplasm. Some of the confluent demyelinating lesions showed a rim of activated macrophages and microglia (Fig. 2f) with early active demyelinating activity at the border and late active demyelinating activity in the lesion center. Demyelination was associated with relative preservation of axons, however, axonal spheroids were numerous particularly at the edge of the lesions (Fig. 2e). Profound microglia activation was also seen in the periplaque white matter (Fig. 2f, g). Within the lesions, a high number of TPPP/p25-positive/MOG-negative oligodendrocytes were visible (Fig. 2h), but remyelination was absent. Perivenous demyelination was also present in the cortex and focally in deep grey matter nuclei. In the cortex, small intracortical (Fig. 2i, j) and single subpial lesions were visible. Reactive protoplasmic astrocytes including Creutzfeldt–Peters cells were found at sites of active demyelination. Active demyelination was associated with deposition of activated complement (C9neo antigen) (Fig. 2k). Inflammatory reaction was relatively mild, likely due to the prior intensive immunotherapy, and consisted of moderate perivascular and mild parenchymal infiltrates of CD4 (Fig. 2l, m) and CD8-positive T-cells (Fig. 2n). In addition, few perivascular CD20 and CD79a positive B-cells were seen (Fig. 2o) and scattered apoptotic leukocytes were visible.

### Autopsy 2

A 52-year-old previously healthy woman presented with 2 weeks of headache and aphasia. Neurologic examination revealed agitation, disorientation, and memory dysfunction. Brain MRI showed multiple cortico-subcortical contrast enhancing lesions with central hemorrhagic transformation in both hemispheres. Extensive infectious workup was negative. CSF analysis revealed 124 white blood cells (predominantly lymphocytes), elevated protein (112 mg/dl), but no oligoclonal bands. Intravenous methylprednisolone was administered but the patient did not respond. Within one week she developed massive brain edema refractory to escalating steroid doses and osmotic therapy, ultimately resulting in brain herniation and death. The time from disease onset to death was 3 weeks. Serum MOG antibodies were confirmed after death of the patient at the Medical University of Vienna (Titer, 1:320). Given the atypical presentation the sample was retested at the Mayo Clinic Neuroimmunology Laboratory, which also confirmed seropositivity (Titer, 1:20). AQP4 antibodies were negative.

**Fig. 3:**
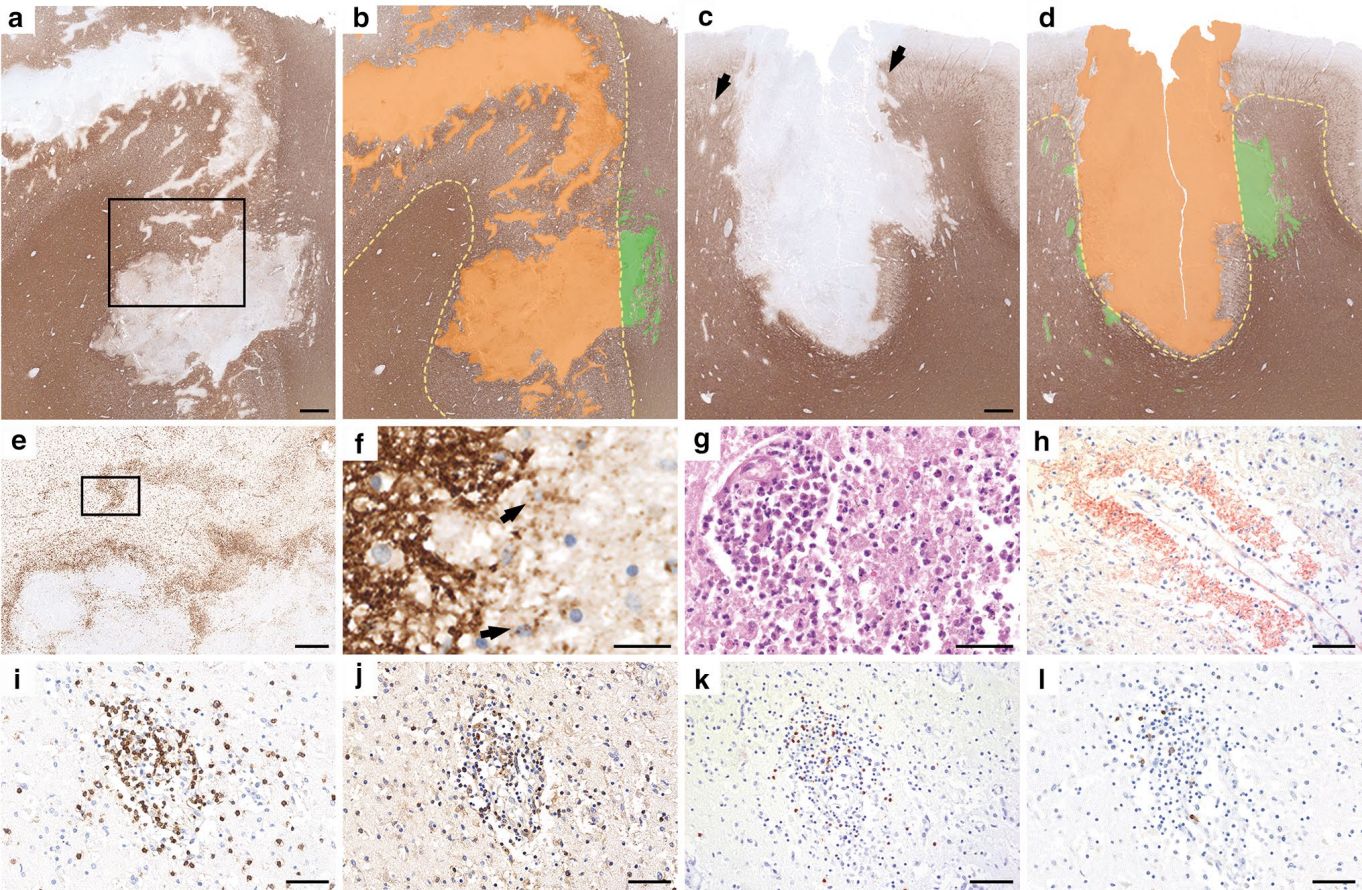
Neuropathology of autopsy case 2. Histopathology of autopsy case 2 shows predominantly cortical plaques that extend into the subcortical white matter (**a–d**, MOG; **b** and **d** schematic drawings, cortical demyelination: orange; white matter demyelination green;) and form multiple perivenous areas of demyelination in the adjacent grey and white matter (**c**, arrows). Activated microglia and macrophages form a rim at the lesion borders and show a perivenous accentuation (**e**, HLADR; rectangle in **a** enlarged in **e**) with MOG-positive demyelination products within the macrophages (**f**, MOG; arrows; rectangle in **e** enlarged in **f**). Some lesions show superimposed ischemic damage with tissue necrosis with massive infiltration of neutrophilic granulocytes (**g**, H&E). Profound perivenous deposition of activated complement complex is visible (**h**, C9neo antigen). The inflammatory infiltrates mainly contain CD3 (**i**) and CD4 positive T cells (**j**), less CD8-positive T cells (**k**) and only few perivascular CD79a positive B cells (**l**). Scale bars **a–d** 1.2 mm; **e** 600 μm; **f** 30 μm; **g–l** 60 μm

Postmortem examination of the brain (Fig. 3) revealed multiple cortico-subcortical demyelinating lesions in the frontal, parietal and temporal lobe (Fig. 3a–d). The demyelination was predominantly confined to the cortex and comprised subpial, intracortical and leukocortical plaques (Fig. 3a–d). In addition, multiple perivenous areas of demyelination occurred in the adjacent grey and white matter (Fig. 3a–d). The lesion borders were characterized by numerous activated microglia and macrophages (Fig. 3e) with early active demyelinating activity (Fig. 3f). Some lesions showed superimposed ischemic damage with tissue necrosis and massive infiltration of neutrophilic granulocytes (Fig. 3g) and loss of astrocytes and axons. These necrotic areas were negative for bacteria or fungi. Active demyelination was associated with massive deposition of activated complement complex (C9neo antigen) (Fig. 3h). The inflammatory infiltrates in the demyelinated plaques mainly contained CD4 positive T cells (Fig. 3i, j), less CD8-positive T cells (Fig. 3k) and only few perivascular CD20 and CD79a positive B cells (Fig. 3l).

## Clinical, serologic and radiologic characteristics of 22 brain biopsy MOGAD patients

Twenty two patients had only a brain biopsy performed (frontal 9; temporal 2; parietal 2; cerebellum 1; fronto-parietal 1; parieto-occipital 1; thalamus 1; brain [not otherwise specified] 5). All 22 patients had an inflammatory central nervous system disorder with detailed clinical information available in 18 (82%). The median age at neurologic symptom onset was 10 years (range, 1–66) and 12 of 22 (55%) were children (< 18 years). In those with exact dates available, the median time from disease onset to biopsy was 7 months, (0–516). The median duration from symptom onset to last follow-up was 43 months (range, 3–516). The demographics, clinical presentation and course, cerebrospinal fluid (CSF) findings and MRI details are summarized in Table 1. Representative MRI scans of biopsied cases are shown in Fig. 1(b–d). The median MOG-IgG titer at Mayo Clinic was 1:100 (range, 1:20–1:10,000) and at the University of Vienna was 1:2560 (range, 1:80–1:5120). Among 14 patients with serial samples, twelve relapsing patients had persistent MOG Ab seropositivity, whereas two patients with a monophasic course were transiently seropositive.

**Table 1:** Clinical, laboratory, and neuroimaging characteristics MOGAD patients that underwent biopsy

	Number of patients (%)	Median (range)^a^
Demographics		
Female sex	13 (59%)	
Age at onset in years	10 (1–66)	
Detailed clinical Information available	18 of 22 (82%)	
Initial clinical manifestation (18 with clinical details)		
ADEM-like brain presentation	11/18 (61%)	
Isolated transverse myelitis	2/18 (11%)	
Isolated optic neuritis	2/18 (11%)	
NMOSD	1/18 (6%)	
Other^a^	2/18 (11%)	
Clinical course, disability and follow-up		
Relapsing	14 of 18 (78%)	
Monophasic	4 of 18 (22%)	
Median # of attacks in those relapsing		5 (2–16)
Types of attacks in those relapsing		
Optic neuritis	32 attacks	
ADEM-like Brain presentation	28 attacks	
Transverse myelitis	11 attacks	
EDSS at last follow-up		2 (1–10)
Duration of follow-up (months)		43 (3–516)
CSF findings		
Elevated white cell count (> 5/μl)	8/13 (62%)	177.5 (28–478/μl)
Elevated protein	8/13 (62%)	
Elevated oligoclonal bands	1/12 (8%)	
MRI brain		
Fluffy-poorly demarcated (ADEM-like)	12/16 (75%)	
Deep grey matter lesions	14/16 (88%)	
Infratentorial lesions	11/16 (69%)	
Parenchymal enhancement	15/16 (94%)	
Leptomeningeal enhancement	5/16 (31%)	

*ADEM* acute disseminated encephalomyelitis, *CSF* cerebrospinal fluid, *EDSS* expanded disability status scale score, *NMOSD* neuromyelitis optica spectrum disorder

^a^Brainstem and myelopathy, 1; progressive encephalitis that resolved with steroids, 1

Among 14 patients with details available, treatments utilized acutely included one or more of: intravenous steroids 13; plasma exchange 7; and intravenous immune globulin (IVIg) 6. All 14 treated patients showed some improvement after acute treatment. Eleven of 12 patients with details available received one or more maintenance immunotherapies or immunomodulatory medications including: oral prednisone 7; rituximab 6; mycophenolate 3; azathioprine 2; IVIg 2; cyclophosphamide 2; glatiramer acetate 2; interferon-β1a 2; ocrelizumab 1; natalizumab 1; or fingolimod 1.

## Pathologic features of 22 biopsied MOGAD patients

**Fig. 4:**
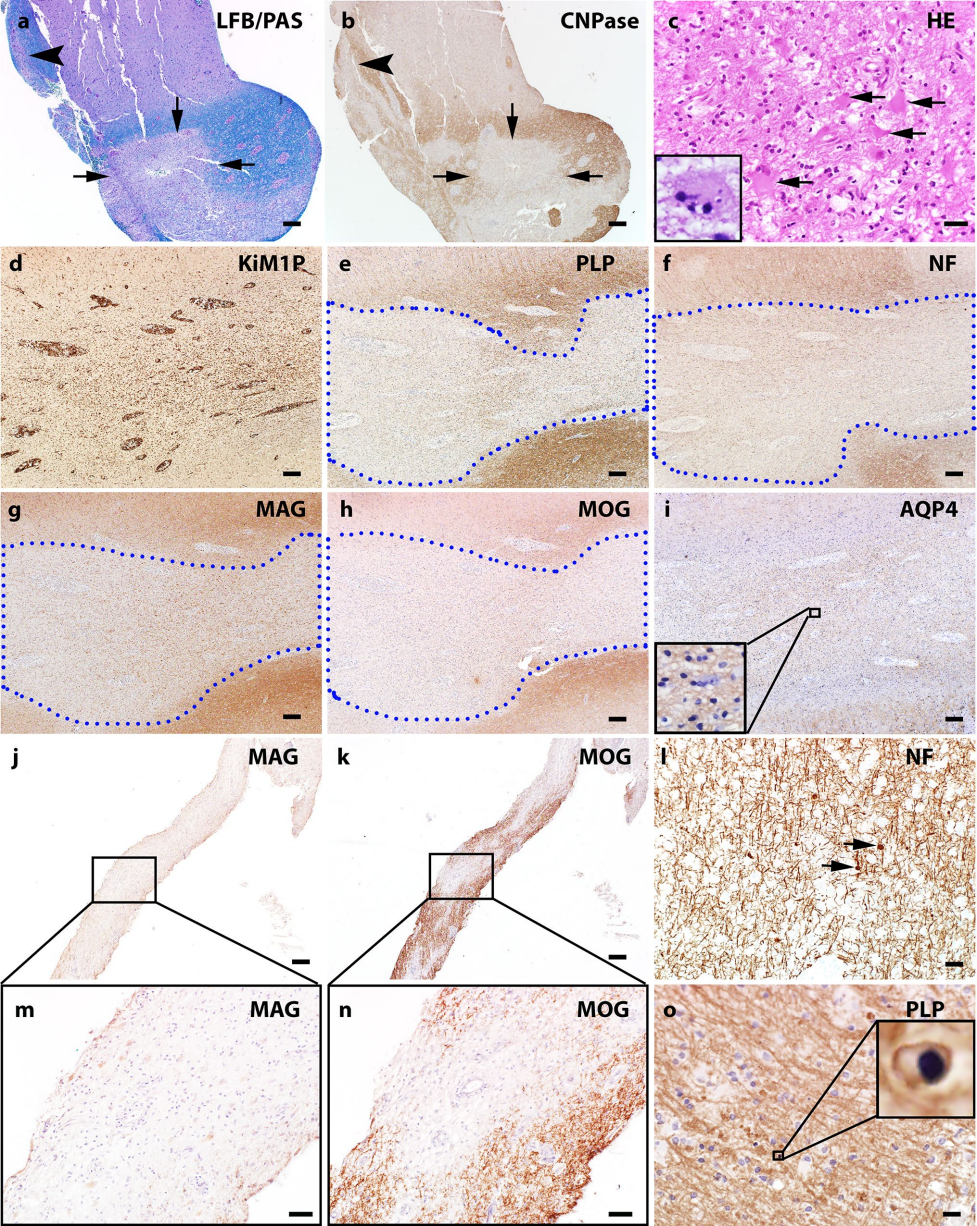
White matter pathology of MOG Ab positive inflammatory demyelinating disease. **a** LFB/PAS stain and myelin protein CNPase immunohistochemistry (**b**) on consecutive sections indicate the perivenous (arrowhead) and confluent (arrows) demyelination that coexists in the subcortical white matter of a MOGAD biopsy. **c** H&E stain shows marked hypertrophic reactive astrocytes present in the white matter lesion and Creutzfeldt-Peters cells are occasionally noted (inset). **d–i** Consecutive sections: **d** KiM1P immunohistochemistry indicates extensive microglia/macrophage infiltration in the white matter with no obvious border. The blue dotted lines contour the demyelinating lesion **e** with relative preserved axons **f**. The loss of minor myelin protein MAG (**g**) and MOG (**h**) are equal. AQP4 is preserved in the lesion **i**. **j** and **k** consecutive sections. Preferential MAG loss (**j**, **m**) with relative MOG preservation (**k**, **n**) is seen in a single MOGAD case. **l** Mild axonal damage characterized by axonal spheroid (indicated with arrows) is present in the demyelinating lesions. **o** Apoptotic oligodendrocytes with condensed nucleus (highlighted in the inset) are seen in the lesions. Scale bars in **a, b, d–k** = 200 μm. Scale bars in **c, l** and **o** = 20 μm

**Fig. 5:**
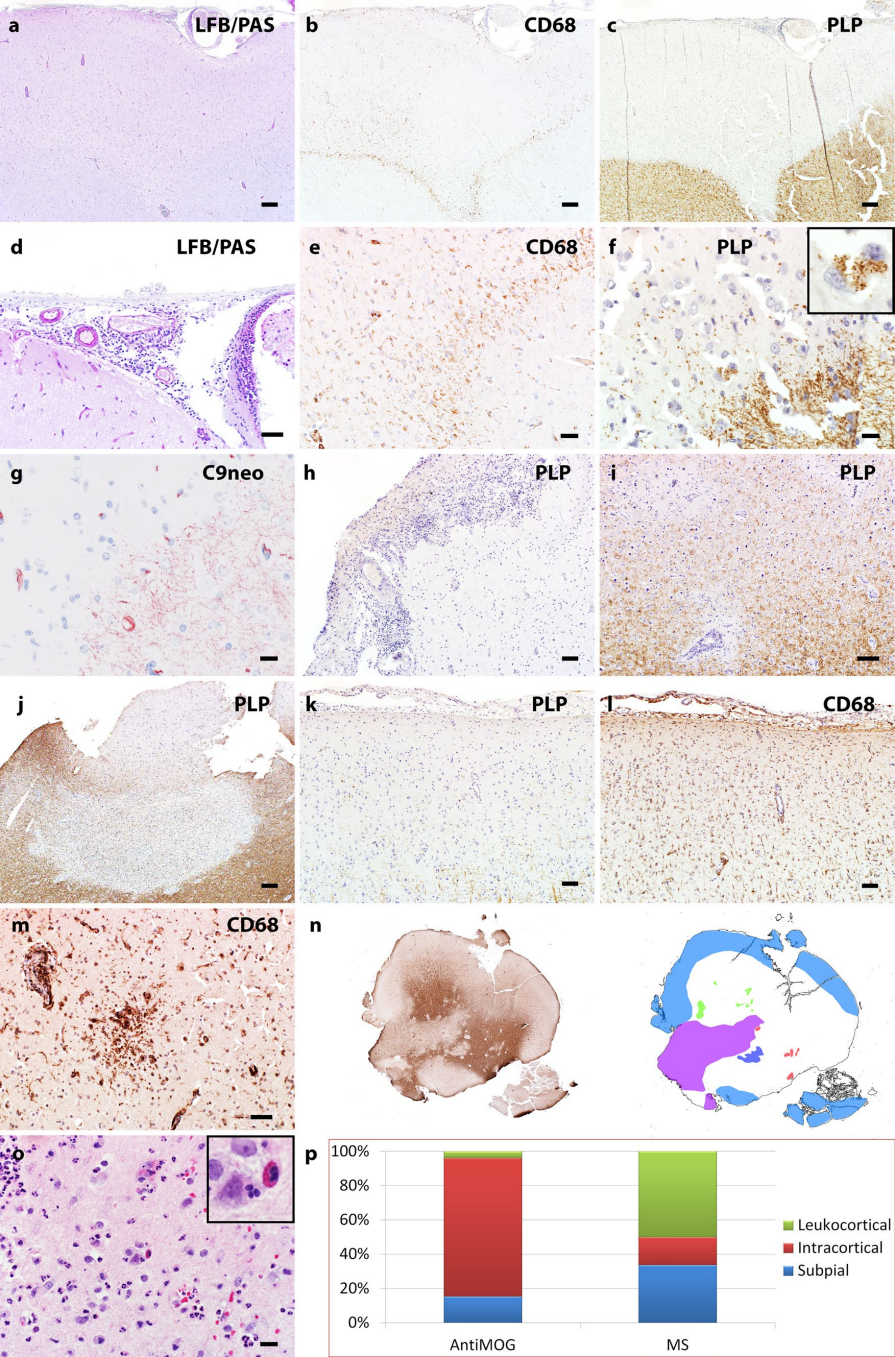
Cortical pathology of MOG-antibody positive inflammatory demyelinating disease**.a–g** Consecutive sections. **a** LFB/PAS staining of the MOGAD cortical biopsy indicates pale cortical staining and meningeal inflammation (the high power image in panel **d** highlights the meningeal infiltration). **b** CD68 immunohistochemistry on the consecutive section to **a** shows microglia/macrophage reactivity in the cortex. The higher power image of **b** (panel **e**) highlights microglia/macrophage reactivity at the border of a cortical lesion. **c** PLP immunohistochemistry indicates extensive cortical myelin loss. **f** High power image shows demyelinating activity at the lesion border characterized by myelin debris laden macrophages (inset). **g** C9neo indicates complement deposition on the myelin fibers in the cortex. **h** PLP immunohistochemistry with hematoxylin counterstain highlights prominent meningeal inflammation associated with extensive subpial cortical demyelination. Focal intracortical (**i**) and leukocortical (**j**) lesions are also present in the MOGAD biopsies. In a subpial cortical lesion (**k**), extensive cortical microglial reactivity is marked (**l**). Microglial aggregates are occasionally seen in the cortex (**m**). **n** PLP staining scan and corresponding schematic figure highlights the complexity of the cortical lesions in a MOGAD case. Light Blue filled square, subpial demyelination. Green filled square, intracortical demyelination; violet filled square, confluent white matter demyelination; red filled square, perivascular white matter demyelination; dark blue filled square, coalescent white matter demyelination. **o** HE stain highlights eosinophils and neutrophils in the cortex. **p** The comparison figure shows the different ratios of cortical lesion patterns between MOGAD and multiple sclerosis. Scale bars in **a, b, c, h, j, k, l** = 200 μm. Scale bars in **e, f, g, o** = 20 μm. Scale bar in **i** = 100 μm. Scale bar in **m** = 50 μm

**Fig. 6:**
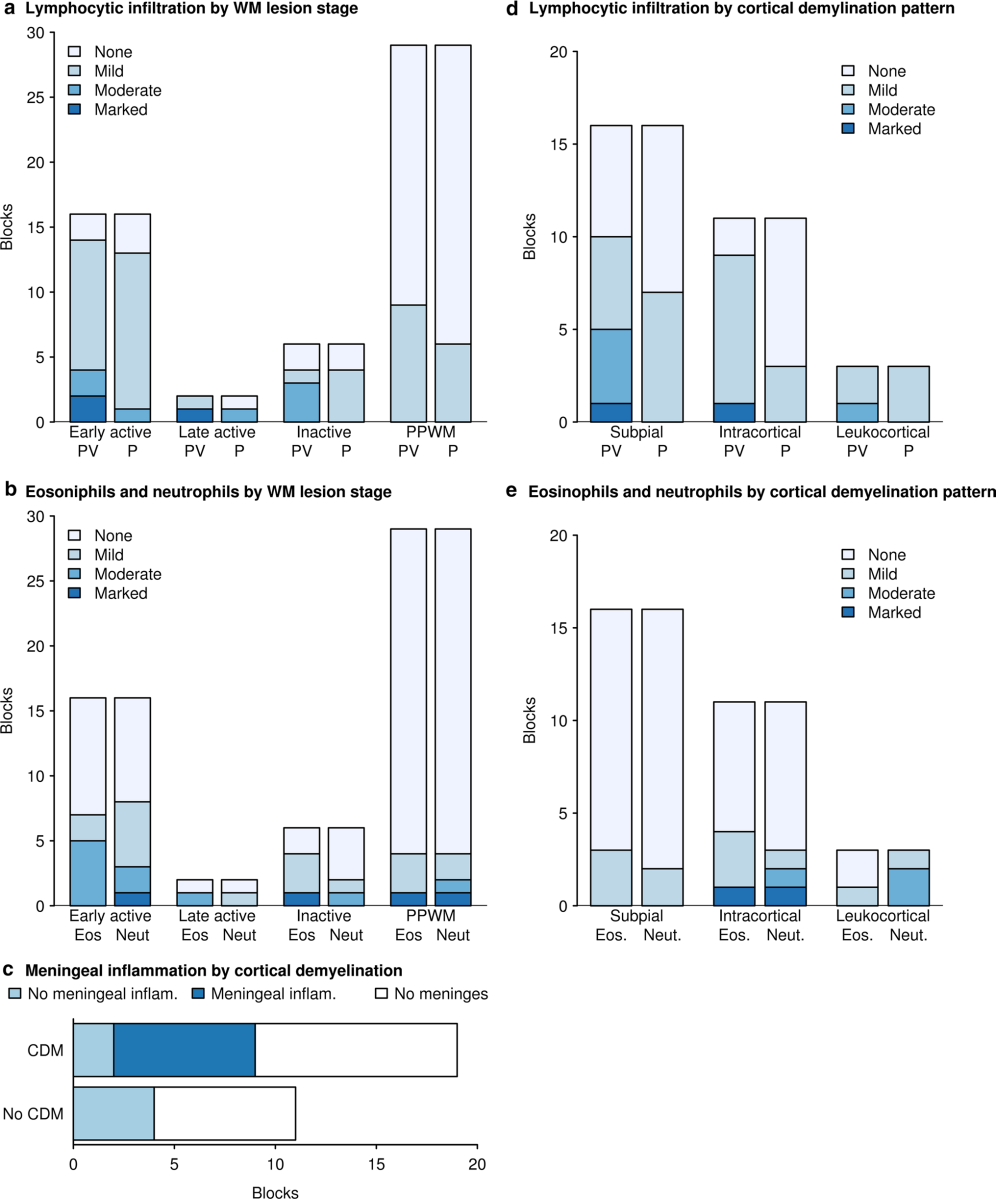
Association between inflammations and demyelination in MOGAD. **a** The extent of lymphocytic infiltration relative to white matter demyelinating stage for both perivascular (PV) and parenchymal (P) infiltration. Early active and late active demyelinating lesions shows more obvious lymphocytic infiltration in terms of severity and frequency compared to non-demyelinated periplaque white matter (PPWM). **b** Eosinophil (Eos) and neutrophil (Neut) infiltration is more prominent in white matter lesions compared to the PPWM. **c** Meningeal infiltration is only present in tissue blocks with cortical demyelination. **d** Both perivascular and diffuse parenchymal lymphocytic inflammation are present in cortical demyelinating lesions. Perivascular inflammation tends to be more prominent. **e** Eosinophils and neutrophils are present in all cortical lesion subtypes, but more often present in intracortical lesions

Among biopsied cases, the available tissue was distributed as follows: 8 cases had white matter tissue only, 2 had cortex only, 11 had both white matter and cortex, and 1 case had white matter and deep gray matter tissue. Of the total of 20 biopsy cases with white matter tissue available for analysis, 6 cases showed no white matter lesions while 14 showed white matter demyelinated lesions. Multiple stages of white matter demyelination could be present within a given person. Eleven biopsies showed early active demyelination, 1 showed late active demyelination, and 4 showed inactive demyelination. The full spectrum of pathological features observed in the MOGAD autopsies was represented in the biopsy cohort, and summarized in Table 2. Representative examples of biopsied white matter and cortex are illustrated in Figs. 4 and 5, respectively. MOGAD biopsy pathology was dominated by a transitional pattern with perivenous and confluent white matter demyelination seen in 50% of the biopsies and a higher incidence of intracortical demyelinated lesions (64% of biopsies), compared to that seen in MS patients with similar disease duration. Abundant myelin-laden macrophages were present within active demyelinating white matter lesions, but the accumulation of activated microglia at the lesion border, typical of slowly expanding / smoldering white matter MS lesions, was not observed. Within the cortex, microglial infiltration often extended beyond the cortical demyelinating lesion (Fig. 5l) with cortical microglial aggregates seen in one biopsy (Fig. 5m). Variable perivascular and parenchymal lymphocyte infiltration was present in the PPWM as well as in all demyelinating stages, most prominent among early active lesions (Fig. 6). In contrast to MS, [49, 71], lymphocyte infiltrates were dominated by CD4+ T-cells, with a median perivascular CD4/CD8 ratio of 2.89 (range, 1.26–29). All cortical demyelinated lesions contained perivascular and parenchymal lymphocytic infiltrates, most prominent within intracortical demyelinated lesions (Fig. 6). In biopsies containing meninges, meningeal inflammation was topographically associated with the presence of cortical demyelination (Fig. 6). A low to moderate number of granulocytes (both eosinophils and neutrophils) were present across demyelinating stages, including in the PPWM, as well as in cortical demyelinated lesions, most pronounced within intracortical demyelinated lesions (Fig. 6).

**Table 2:** Comparison of histopathological features in MOGAD autopsy and biopsy cohort

Pathological feature	Present in autopsy (±)	Present in biopsies (±)	Frequency in Biopsy Cohort (%)
Plaque location			
White matter	+	+	14/20 (70%)
Cortex	+	+	10/13 (77%)
Deep Gray Matter	+	+	1/1 (100%)
White matter demyelination pattern			
Perivenous	+	+	3/14 (21%)
Confluent	+	+	4/14 (29%)
Transitional (perivenous + confluent)	+	+	7/14 (50%)
White matter plaque type			
Chronic active with sharp macrophage border	–	–	0/20 (0%)
Slowly expanding/smoldering	–	–	0/20 (0%)
Cortical plaque type			
Leukocortical	+	+	1/13 (8%)
Intracortical	+	+	9/13 (69%)
Subpial	+	+	8/13 (62%)
Inflammatory features			
Lymphocytes	+	+	20/22 (91%)
Granulocytes	+	+	17/22 (77%)
Meningeal Inflammation	+	+	6/7 (86%)
Complement Deposition (Pattern II)	+	+	8/8 (100%)
Complement deposition glial limitans	–	–	0/8 (0%)
Comparative Myelin Immunohistochemistry			
MAG loss > MOG loss (pattern III)	–	+	1/8 (13%)
MOG loss > MAG loss	–	–	0/20 (0%)
Cellular features			
Oligodendrocyte apoptosis	+	+	9/20 (45%)
Axonal spheroids	+	+	7/13 (54%)
Axonal loss	+	+	1/13 (8%)
Astrogliosis	+	+	21/22 (95%)
Creutzfeldt-Peters cell	+	+	3/22 (14%)
Dystrophic astrocytes	–	–	0/22(0%)
AQP4 loss	–	–	0/17 (0%)

Variable denominators are based on number of biopsies containing specific region analyzed for a given feature (i.e., white matter, cortex), available staining, or feature classification based on specific stage of demyelinating activity (i.e., immunopattern)

Immunopattern classification was interpretable among 8 of 11 early active biopsies. Complement deposition (C9neo antigen) within macrophages and on myelin sheaths, typical of pattern II MS, was present in 8/8 active biopsies, including a single biopsy characterized by an overlap of immunopattern II and pattern III, defined by a preferential loss of myelin associated glycoprotein (MAG) and oligodendrocyte apoptosis (Fig. 4j, k, m, n). Axons were generally intact with few axonal spheroids apart from a single biopsy (Fig. 4l). AQP4 was preserved in all cases and dystrophic astrocytes were absent.

## Discussion

MOGAD pathology is characterized by the coexistence of perivenous and confluent primary demyelination with partial axonal preservation and reactive gliosis in the white and gray matter, with particular abundance of intracortical demyelinating lesions. This occurs on the background of CD4-dominated T cell and granulocytic inflammatory infiltrates. Contrary to classical AQP4-IgG seropositive NMOSD, in MOGAD the expression of AQP4 is preserved.

As described already in the earliest reports of MS pathology [40], the demyelinated lesions arise around focal perivenous inflammatory aggregates as acute plaques, which further expand by a zone of lesional activity surrounding the inactive core (chronic active lesions; [36]). A subset of these chronic active MS lesions are smoldering plaques with a rim of activated microglia at the edge and very sparse macrophages with early myelin degradation products [20, 36], which enlarge slowly over months or years [2, 10]. Thus, chronic active focal white matter lesions are typical for multiple sclerosis pathology and present at all disease stages [20]. However, with the exception of a single lesion with a macrophage rim in a single case (Fig. 3a, e), slowly expanding demyelinated plaques are absent in our current case series of MOGAD cases. The MOGAD cases show an ADEM pattern of demyelination, characterized by widespread and diffuse inflammation and demyelination around small venules, which in some instances form confluent plaques by fusion, but not by radial expansion [81].

The coexistence of both perivenous and confluent demyelination seen within our MOGAD cohort was previously described in a subset of pathologically defined ADEM [80, 81], where it was associated with a greater risk of relapse. Although these may have been cases of MOGAD (30–50% of clinical ADEM cases are MOG-IgG seropositive) [63], the absence of available sera to confirm a diagnosis in this published series precludes making any definitive conclusions and makes comparisons difficult to prior cohorts, in which serological testing was not undertaken.

Cortical demyelination is common among the MOGAD cohort, and, as observed in MS, topographically associated with meningeal inflammation [47]. In addition, subpial cortical demyelination, similar to that seen in MS, is present. Thus subpial demyelination is present only in MS, ADEM, and MOGAD, while it is not found in any other inflammatory, demyelinating, metabolic or neurodegenerative disease studied up to now [17, 52]. However, in contrast to MS, intracortical rather than leukocortical demyelinated lesions predominate. Although two prior MOGAD biopsied cases reported cortical encephalitis without demyelination, this may have potentially been due to a very early stage of the disease process or an effect of treatment prior to biopsy [21, 26].

The CD4-dominated inflammatory infiltrate in MOGAD differentiates this disease from MS, in which inflammatory infiltrates are mainly composed of CD8+ T-cells, as shown in large cohort studies including all disease stages [49, 71]. Higher numbers of CD4+ T-cells in MS cases have so far only been described in single, atypical cases [61]. Furthermore, in MS there are high numbers of B-cells in the inflammatory infiltrates and the CSF, as well as a profound further differentiation of B-cells into plasma cells, which produce antibodies, reflected in the high frequency of oligoclonal bands in MS. This is particularly the case in patients with fulminant disease courses, where B-cells even outnumber T-cells in the lesions [49]. Given that one of the MOGAD autopsies and some MOGAD biopsies were treated with B cell depleting agents (rituximab or ocrelizumab), we are unable to specifically comment on the ratio of B cells and T cells in the lesions. However, given the low frequency of oligoclonal bands in MOGAD, it suggests that most of the pathogenic antibodies (as in AQP4-IgG seropositive NMOSD), come from the blood and only a small proportion from local intrathecal synthesis.

Both autopsies, and all 8 biopsy cases that could be reliably immunopattern classified, demonstrate evidence of complement deposition within active white matter lesions, consistent with prior reports [12, 27]. This is not surprising as MOG-IgG is of IgG1 isotype and capable of activating complement. Although a perivascular pattern of complement deposition is seen in the autopsy cases, the absence of complement deposition on astrocytes of the glia limitans is distinct from AQP4-IgG seropositive NMOSD pathology, where the complement deposition colocalizes to sites of AQP4 expression. These findings suggest an antibody-mediated mechanism in MOGAD, and this view is supported by experimental studies, showing the induction of demyelination by patient derived anti-MOG antibodies after direct injection into the brain tissue [64] or after their injection into the cerebrospinal fluid of animals with T-cell mediated autoimmune encephalomyelitis [68]. However we do not observe a selective loss of the MOG protein, which would only be seen if the antibody strips the outer myelin lamella, leaving the compact myelin intact. This does not occur even in the experimental models of MOG-EAE. It is plausible that when the antibody binds to the outer myelin surface, the myelin is either completely destroyed by complement or by antibody dependent cellular cytotoxicity (ADCC) phagocytosis. A selective loss of the MOG antigen would only occur when the binding of the MOG-antibody leads to endocytic internalization of the antigen into a cell, which still remains viable, albeit functionally compromised. This is a major mechanism by which AQP4-IgG targets the AQP4-antigen [42].

A single MOGAD biopsy case demonstrated an overlap of immunopattern II and III, with the presence of complement deposition, as well as MAG loss. The variable destruction of oligodendrocytes seen in MOGAD pathology, with evidence of oligodendrocyte apoptosis, could contribute to MAG loss, as this occurs in the setting of metabolic abnormalities in the oligodendrocyte unable to maintain its distal periaxonal cell processes, leading to a dying back oligodendrogliopathy [48]. Therefore, MAG loss is not specific to MS, but rather seen in a number of disorders including stroke, progressive multifocal leukoencephalopathy, and MS [1]. Monoclonal MOG Ab 818C5 induces β-tubulin dephosphorylation, retraction of oligodendrocyte processes and alters oligodendrocyte cytoskeletal structure, therefore it is plausible that a humoral process in MOGAD targeting oligodendrocytes could explain the observed overlap of immunopattern II and III in this single biopsy case [11, 16, 50].

The clinical characteristics of MOGAD biopsied patients in this study are consistent with what has been reported in the literature of MOGAD with similar demographics (median age of 10 years, similar proportion of males and females), characteristic clinical phenotypes (ADEM-like presentation and optic neuritis dominating, followed by transverse myelitis and NMOSD), typical CSF findings (a high median white cell count [> 100/µL] and low frequency of oligoclonal bands [< 10%]), clinical course (optic neuritis dominating) and overall good long-term outcome in most biopsied cases (median EDSS of 2 at last follow-up). The autopsy cases also have features that fall within the realm of what has been reported with MOGAD, with seizures and encephalopathy being recognized manifestations, fulminant cases previously reported, and a predominantly cortical encephalitis (as in autopsy 2) [13, 22, 54]. The white and gray matter lesions on MRI and absence of CSF oligoclonal bands in the autopsied cases is also typical [63]; however, the hemorrhagic transformation and fulminant course resulting in brain herniation in autopsy case 2 is atypical and may relate to the concomitant ischemic changes. Confirmation of the MOG-antibody seropositivity in 2 independent laboratories however ensures this was a case of MOGAD.

Heterogeneity in age at onset, disease course (monophasic versus relapsing), therapies utilized, clinical course, pathology and antibody titers in MOGAD patients in this study is consistent with the literature on MOGAD [34], but clear differences remain from MS and AQP4-IgG-seropositive NMOSD that suggest MOGAD is a distinct entity. The wide range in age of onset of MOGAD is similar to what is observed in other defined subsets of CNS inflammatory demyelinating diseases (both MS and aquaporin-4-IgG seropositive NMOSD can impact children or the elderly) [60, 73, 74]. There are however unequivocal major differences between MOGAD and MS in presenting manifestations (ADEM is common in MOGAD but very rare in MS), clinical course (a progressive course is not reported with MOGAD but the majority of MS patients eventually develop a progressive course) [30], frequency of CSF oligoclonal bands (8% of MOGAD in our study [similar to prior reports] [15, 34] versus MS where it occurs in 88%) [14], and there are a number of MRI differences in the spinal cord, brain and optic nerve that have been reported [7, 15, 33]. The response to treatments is difficult to determine in this study as well as prior studies of MOGAD, given their retrospective nature, the heterogeneity of treatments used and the lack of a control group. There is an urgent need for prospective randomized placebo-controlled trials in MOGAD, which may give additional insight into the underlying pathophysiology.

The serologic detection of MOG-IgG is useful diagnostically and the features of the antibody may provide insight into pathogenesis. A recent large study of MOG antibodies in MS showed it is detected in only 0.2%, highlighting its utility in discriminating from MS [8]. While in our study we used two sites for MOG-IgG detection, our multi-center collaborative studies have shown the live-cell based MOG assay cut-offs show consistent results across centers for a non-MS phenotype [77]. The frequency of MOG-IgG and AQP4-IgG coexistence was reported at 0.06% which suggests a distinct immunopathogenesis to MOGAD and AQP4-IgG seropositive NMOSD, rather than MOG-IgG representing an epiphenomenon [37]. The presence of transient seropositivity is associated with a monophasic course in this study and is similar to prior reports [23]. Higher titers have also been associated with an increased tendency towards a relapsing course but evaluation in our study is limited by differing assay cut-offs utilized at the different sites [23, 45].

The majority of MOGAD cases in this series have a relapsing clinical course, and none developed a progressive course at last follow-up. A pathological substrate of progression in MS is the presence of slowly expanding (smoldering) lesions in the white matter [19], which are largely absent in MOGAD. Although our cohort is skewed toward early disease, a secondary progressive course typical of MS rarely if ever occurs in MOGAD [30]. As a subset of smoldering lesions have an iron ring in MRI [2, 10], it would be helpful to determine whether MOGAD patients ever develop iron ring lesions.

From a clinical and radiologic perspective, MOGAD more closely resembles AQP4-IgG seropositive NMOSD, than MS. This is evident by the clinical, radiologic and CSF findings that accompany MOGAD which include a high frequency of bilateral optic neuritis, more severe episodes (frequent blindness/paraplegia), longitudinally-extensive lesions in the optic nerve and spinal cord, lesions adjacent to the 3rd ventricle, central rather than peripheral spinal cord lesions, CSF white cell count often > 50 µL and CSF unique oligoclonal bands rarely detected [15, 29, 33]. However this study shows, despite this clinico-radiologic overlap, the pathology of MOGAD is distinct from AQP4-IgG seropositive NMOSD. The presence of hypertrophic reactive astrocytes, preserved to increased AQP4 expression [67, 82], occasional Creutzfeldt cells and frequent cortical demyelination in MOGAD, are helpful pathologic discriminators from AQP4-IgG seropositive NMOSD, which is characterized by AQP4 loss, dystrophic astrocytes, and absence of cortical demyelination [58, 59]. Our findings support most prior MOGAD pathology cases which similarly reported no AQP4 loss [12]. However, two MOGAD pathology cases reported variable AQP4 loss [66] and a single case with dual MOG-IgG and AQP4-IgG seropositivity also reported AQP4 loss [13].

The pathogenic significance of human anti-MOG antibodies in the induction of demyelination has been shown in several recent experimental studies [55, 64, 68]. Although the pathophysiological mechanisms of MOG-antibody associated inflammatory demyelinating disease in humans are so far not fully established, they have been extensively characterized in experimental models of MOG induced experimental autoimmune encephalomyelitis (MOG-EAE). Circulating MOG antibodies alone do not induce inflammation, demyelination and neurodegeneration in normal rodents [43]. However, MOG antibodies, when directed against a conformational epitope, expressed on the surface of oligodendrocytes and myelin sheaths [4], may trigger demyelination in vitro or in vivo after injection into the cerebrospinal fluid or the central nervous system (CNS) tissue, but require the presence of complement or of cytokines that induce macrophage activation and antibody dependent cellular cytotoxicity [44, 72]. In vivo, circulating anti-MOG antibodies become pathogenic when they reach the brain in the context of inflammation, mediated through MHC Class II restricted CD4+ T-lymphocytes [43]. The pathology in the brain and spinal cord in co-transfer models of encephalitogenic T-cells and anti-MOG antibodies depend upon the quantitative balance between T-cell mediated inflammation and the demyelinating antibodies. Thus, a massive T-cell mediated inflammation in the presence of circulating anti-MOG antibodies results in an ADEM-like inflammatory disease, with perivenous (sometimes confluent) demyelination, while in the presence of a mild T-cell mediated inflammation with high titers of circulating antibodies focal (MS-like) demyelinating plaques are induced [41].

Experimental demyelination by anti-MOG antibodies in the context of T-cell mediated inflammation is accomplished either through complement activation or through antibody dependent cellular cytotoxicity. The extent of complement activation in the lesions depends upon the isotype of the antibodies, but demyelination may also be induced by anti-MOG antibodies with poor complement activation properties through interaction with activated effector cells, such as macrophages [56, 57]. In addition, anti-MOG antibodies can also enhance CD4+ T-cell mediated inflammation through augmented antigen presentation, when inflammation is induced by T-cells recognizing MOG [18]. When inflammation is induced by T-cells directed against other CNS antigens, MOG antibodies trigger demyelination, but do not augment the T-cell mediated inflammation. These data, obtained with monoclonal rodent antibodies, have recently been confirmed in experimental transfer studies using patient derived anti-MOG antibodies [68].

MOG-EAE induced by active sensitization with full length recombinant MOG has shown that the clinical presentation and the topographic distribution of lesions in the central nervous system depend upon the genetic background of the animals. Thus, MOG-EAE in Lewis rats mainly induces an inflammatory disease with limited perivenous demyelination, in BN rats an NMOSD like phenotype, in DA rats large confluent demyelinated lesions in all brain regions and in Wistar rats an inflammatory demyelinating pathology with prominent induction of cortical lesions [69, 70]. Fine mapping of gene regions, associated with these different disease phenotypes, identified genes located in the major histocompatibility complex (MHC) region as the main culprits. However, this does not necessarily mean that these phenotypes are related to differences in antigen presentation, since additional genes such as the MOG gene itself or immune effector genes are also located within this region of the genome [78]. Since MOG is expressed on mature oligodendrocytes, but not on oligodendrocyte progenitor cells, rapid recruitment of new oligodendrocytes occurs in the lesions, which is associated with rapid and complete remyelination [6, 65].

## Conclusion

As the MOGAD biopsies reflect the full spectrum of pathological features seen in the autopsy cases, the presence of either perivenous alone, or an overlap of perivenous and confluent white matter demyelination, in the setting of prominent intracortical demyelination on brain biopsy, as well as an inflammatory reaction in which CD4+ T-cells outnumber CD8+ cells in the presence of granulocytes, and preserved AQP4 expression, should prompt consideration for MOG-antibody testing. The pathology is helpful in differentiating MOGAD from other CNS demyelinating diseases. Experimental studies in MOG-EAE reflect the broad range of the clinical and pathological phenotypes of MOGAD, as described in our present study, and provide potential mechanistic explanations for their occurrence in different patients.
